# Behavioural insights to support increased consumption of quality protein maize by young children: a cluster randomised trial in Ethiopia

**DOI:** 10.1136/bmjgh-2020-002705

**Published:** 2020-12-18

**Authors:** Katherine Donato, Margaret McConnell, Dan Han, Nilupa S Gunaratna, Masresha Tessema, Hugo De Groote, Jessica Cohen

**Affiliations:** 1 Boston Consulting Group, Denver, Colorado, USA; 2 Global Health and Population, Harvard University T H Chan School of Public Health, Boston, Massachusetts, USA; 3 Lee Kuan Yew School of Public Policy, National University of Singapore, Singapore; 4 Public Health, Purdue University, West Lafayette, Indiana, USA; 5 Ethiopian Public Health Institute, Addis Ababa, Oromia, Ethiopia; 6 Socioeconomics Program, CIMMYT Kenya, Nairobi, Kenya

**Keywords:** nutrition, child health, cluster randomized trial

## Abstract

**Introduction:**

Biofortified crops have tremendous potential to improve child nutrition. We tested whether complementing the distribution of quality protein maize (QPM) with a package of interventions informed by behavioural insights could support greater consumption of QPM by young children and translate into improved growth.

**Methods:**

We conducted a cluster-randomised trial in Oromia, Ethiopia. Clusters of households with a child between 6 and 35 months were randomised into an arm receiving QPM seed only (320 households, 203 clusters) or an arm receiving QPM seed and a child consumption targeting intervention (290 households, 183 clusters). The intervention package included tools to help caregivers keep QPM separate from conventional maize and to earmark QPM specifically for child consumption, as well as encouragement regarding cooking QPM specifically for young children. We analysed the impact of the intervention on food storage, cooking and consumption behaviours and on anthropometric measures (weight-for-age, height-for-age z scores).

**Results:**

The consumption targeting intervention increased the probability of child consumption of QPM in the past week by 17.3 percentage points (pp) (95% CI 9.4 pp to 25.1 pp; p<0.01), increased the probability that QPM flour was stored separately from conventional maize by 46.5 pp (95% CI 38.3 pp to 54.7 pp; p<0.01) and increased the probability that caregivers cooked QPM specifically for young children in the past week by 14.4 pp (95% CI 7.9 pp to 20.9 pp; p<0.01). These effects persisted, but were attenuated, 10 months postintervention. No significant effects on anthropometric outcomes were found.

**Conclusions:**

Enhancing the distribution of new, biofortified crop varieties with a consumption targeting campaign can change storage, cooking and consumption behaviours. However, these improved behaviours did not translate into increased growth in this setting.

**Trial registration number:**

NCT02710760 and AEARCTR0000786.

**Key questions**

**What is already known?**

- Children in low-income and middle-income countries are at high risk of undernutrition, a leading cause of under 5 mortality.
- Quality protein maize (QPM) has demonstrated positive impact on child growth in limited, tightly controlled settings.
- Limited research addresses ways to ensure sufficient intake of QPM by young children when scaling up QPM at the population level.

**What are the new findings?**

- An intervention targeting QPM consumption to small children that was informed by insights from behavioural economics led to a significant increase in compliance with recommended QPM storage and cooking behaviours among household caregivers as well as QPM consumption among young children.
- The intervention did not improve child anthropometric outcomes, potentially because the amount of QPM consumed was not enough to substantially improve growth or the study was not long enough to observe changes in growth.

**What do the new findings imply?**

- Behavioural strategies, including those tested in this study, have the potential to elicit increased consumption of biofortified crops among target populations. Future research should continue to pinpoint the relationship between QPM consumption in uncontrolled settings and child growth.

## Introduction

Undernutrition is a primary cause of death for nearly 45% of deaths among children under 5 years in low-income and middle-income countries and is a particularly serious problem in Ethiopia, where two out of five children are stunted.^1 2^ Poor dietary quality is an important cause of malnutrition and is common in Ethiopia^3^ where children’s diets rely on locally produced staple crops like maize, teff, wheat and sorghum.^4^ These foods often have low levels of micronutrients such as iron, zinc and vitamin A, and lack high-quality proteins, which are critical to growth and immune function.^5–8^

Biofortified crops are enhanced with micronutrients and amino acids through agronomic practices, conventional plant breeding or genetic modification, and have been proposed as an important means of enriching dietary quality in agriculture-dependent countries.^9 10^ Several types of biofortified crop varieties have been developed—including iron-biofortified pearl millet^11 12^ and provitamin A-rich orange sweet potato^13–15^—and research into how best to develop and accelerate the adoption of these varieties has been growing recently.^16^ Among these biofortified crop varieties is quality protein maize (QPM), conventionally bred maize varieties enhanced with superior protein quality, ensuring a higher content of the limiting amino acids lysine and tryptophan.^17^

While several studies have determined the acceptability of QPM^18^ and its potential impact on growth in limited, tightly controlled settings,^19^ little is known about policy approaches to ensuring sufficient intake of QPM by young children when disseminating these varieties at the population level. Research on biofortification and child nutrition has largely taken place in settings where feeding and agricultural practices can be tightly controlled.^20–23^ The introduction of fortified food in community settings has faced challenges of adoption and compliance.^24 25^ Even in larger trials of biofortified foods, many households fail to adopt and consume biofortified foods.^13 26^

This paper reports on a randomised controlled trial in Ethiopia, testing whether a package of interventions informed by behavioural insights can lead to increased QPM consumption by children and improved child growth. The intervention was designed to increase the targeting of QPM to young children within the household through the provision of information regarding benefits to small children and through encouraging specific behaviours related to QPM storage, cooking and feeding. The intervention was motivated by previous research in behavioural science, which has shown that behaviourally informed interventions, including labelling and earmarking tools, can induce substantial behaviour change.^27 28^ We tested whether layering these behavioural interventions with traditional public health messaging on top of QPM seed distribution can increase child consumption and growth. Our study provides new evidence about how to better target the potential benefits of the dissemination of biofortified crops in Africa to young children.

## Methods

### Setting

This study took place between April 2015 and June 2016 in the Oromia region of Ethiopia and involved households from 12 kebeles (the smallest administrative unit in Ethiopia consisting of approximately 500 households). About 90% of the population in Oromia live in rural areas. Most households in this region engage in agriculture^1^ and meet food needs largely through home production, with over 60% of own-produced cereals (eg, maize) and pulses (eg, lentils) being used for household consumption.^29^ In Oromia, only 17.8% of children age 6–23 months meet the WHO’s minimum dietary diversity requirements, and 37% of children under 5 are stunted.^1^ Maize is both an important agricultural crop and the primary cereal consumed by Ethiopians.^30^ Many households cook and share communal meals of staple-based foods, such as injera, from one family plate. As part of Ethiopia’s established health extension programme, households are organised into small community health groups consisting of six households, which are designed to facilitate interactions with community health workers, including the sharing of nutritional information.^31^

### Quality protein maize

QPM is a set of conventionally modified maize varieties with enhanced protein quality. It has many characteristics comparable with conventional maize varieties,^32 33^ and has been found to be acceptable to consumers in Ethiopia.^18 34^ To maintain its nutritional traits and the quality of its protein content, QPM must be kept separate from other maize during harvest and storage. Throughout this study, QPM seed was not available in the market, but was disseminated in small demonstration projects throughout the Oromia region by Nutritious Maize for Ethiopia (NuME)—a project administered by the International Maize and Wheat Improvement Centre. The varieties of QPM used in this study were developed over many years, as agriculture and nutritional experts sought to optimise nutritional properties^35^ without compromising on yield or storage quality. Earlier versions of QPM did not perform adequately in terms of yield and resistance to storage pests, and additional breeding was needed to correct for those traits. We designed the study to allow households to adopt a small amount of the new varieties while also allowing them to grow enough to substantially transform the diets of small children who eat relatively less than adults.

### Sample selection, seed dissemination and enrolment

Before the study, NuME organised field demonstrations of QPM varieties suitable for study areas. Both male and female household members in homes with land available for cultivation were invited to attend. An average of approximately 250 people attended each field demonstration. Agricultural extension agents demonstrated how to grow and store QPM in ways that maintain protein quality, illustrated to participants that cooking QPM was the same as cooking conventional maize and emphasised the nutritional benefits of QPM for young children. They recorded the contact information of households that attended, including the ages of all children in the household.

A random sample of households who attended the demonstration projects and who had a child that would be 6–35 months old at the start of the study were selected for potential inclusion in the study from existing lists by a study staff member using a quasi-random number generator.^36^ Among these households, 2/3 were randomly selected to be offered QPM seed and 1/3 served as a pure control group. Agricultural extension workers visited selected households and asked if they would like to order up to three 2 kg bags of QPM seed at no cost and repeated key messages from the demonstration to the household head and caregivers whenever available. Almost all households (95%) chose to order QPM seed, averaging 2.9 bags of QPM seed per household, enough to plant 0.23 ha, approximately one-fifth of the total owned land of the average household.

Households who ordered QPM seed were then further prospectively randomised into the behavioural targeting intervention described below. In other research, we compare outcomes between the control group and households receiving QPM seed,^37^ but this manuscript reports only on the two arms offered QPM in order to examine how the behavioural intervention impacted targeted behaviours including storage, cooking and feeding of QPM and whether these behavioural changes translated into improved growth.

Study eligibility was verified and written informed consent was obtained via written signature or thumbprint at the time of the baseline survey (described below). Households were eligible to participate if they: (1) had at least one child ages 6–35 months at the time of the baseline survey, (2) owned and farmed their own land and (3) did not intend to move over the study period. The child aged 6–35 months at baseline was designated as the ‘index child’ for the purpose of data collection. If there was more than one child in this age range the youngest of the eligible children was chosen. Records documenting reasons for ineligibility were lost during study fielding. The vast majority of participants were ineligible because they did not fall within the eligible age range.

### Intervention design and randomisation

Households that ordered QPM seed were grouped at the community health group level and community health groups were randomly assigned with equal probability into an arm in which households receive no further intervention (‘QPM-only group’) or an arm in which households receive the targeting intervention package (‘QPM+targeting group’). Randomisation was conducted by a study staff member using a quasi-random number generator on a computer.^36^ Randomisation was stratified at the kebele level, and it was clustered by community health group because of the possibility of sharing materials or information within the community health group. While the randomisation was by the community health group cluster, the treatment was at the individual household level. The treatment was not blinded and there was no allocation concealment.

**Figure 1:**
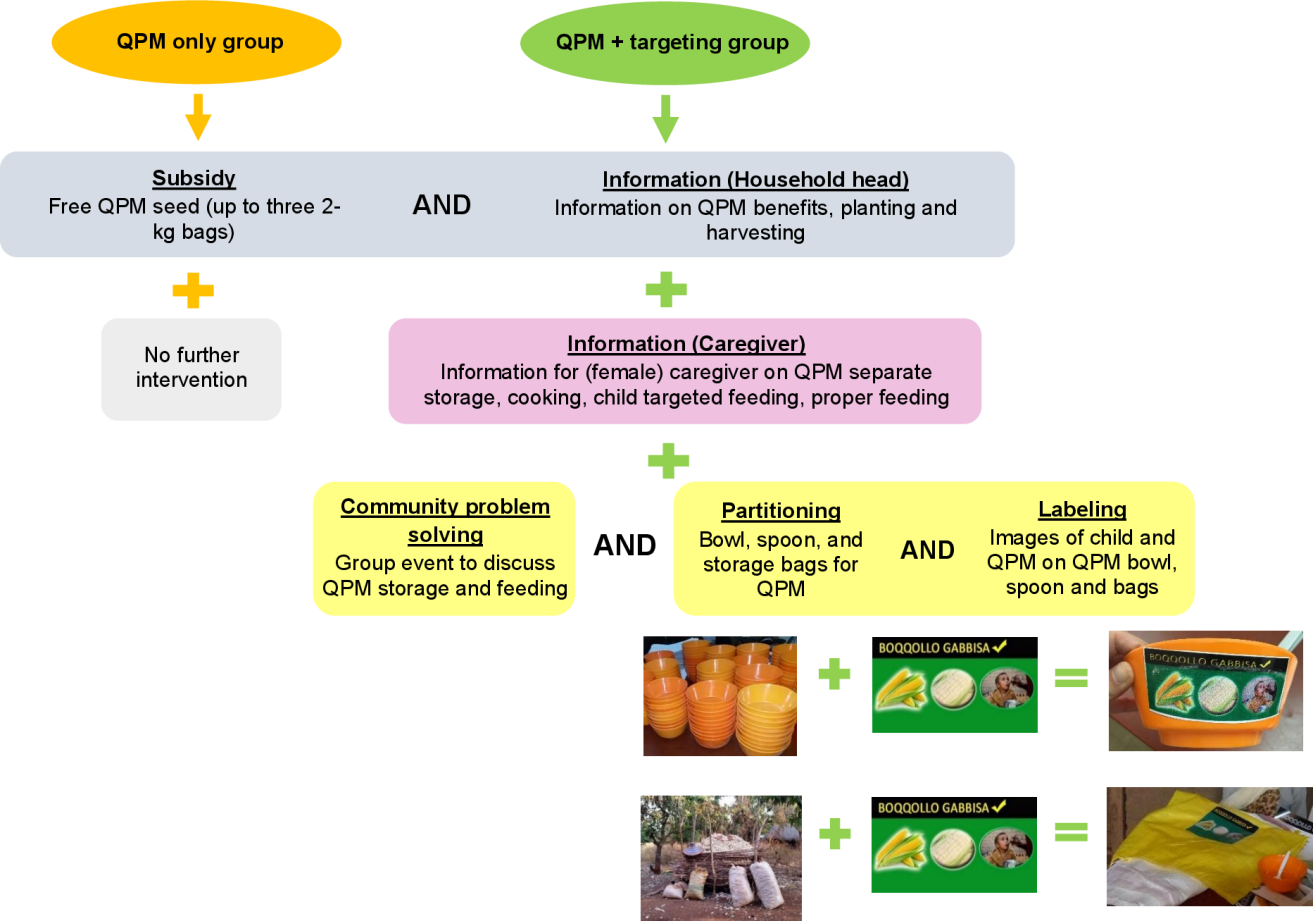
Intervention. QPM, quality protein maize.

The intervention package was designed to target QPM towards consumption by young children using a combination of insights from behavioural economics, traditional public health messaging, and aides to help households follow through with recommended agriculture, cooking and feeding practices (figure 1). First, households were given information about the nutritional benefits of QPM and the importance of targeting QPM to young children following the completion of the baseline survey. Second, households were invited to a group meeting with female caregivers, which reinforced general principles of proper feeding of young children and provided strategies to target QPM towards young children. For example, the intervention suggested one strategy of cooking porridge with QPM, which is nutrient dense, easy for small children to consume and less likely to be consumed by adult household members and therefore better targeted specifically towards young children. Furthermore, at the end of the group meeting, caregivers were given specific grain and flour storage bags to keep the QPM separate from other maize at all stages of production. The bags were marked with a colourful label that had a picture of an infant eating, images of white and yellow maize, and ‘QPM’ written in the local language. This component of the intervention was based on evidence that partitioning resources into smaller units^38^ and putting a salient ‘label’ on those units,^39^ can encourage the use of resources for intended purposes—in this case, the consumption of QPM by young children instead of other household members. The group meeting was conducted by study staff who were employees of the NuME programme. Following the group meeting, households who had been invited but did not attend were visited in their homes to deliver similar messaging. Interventions intentionally engaged female caregivers, who may not always be included in discussions around agricultural topics. Finally, key targeting messages were reinforced after the conclusion of data collection during a follow-up household visit. In households assigned to the targeting intervention female caregivers were including in conversations about QPM whenever possible, even when conversations focused on agricultural properties (such as the need to keep QPM separate from other maize).

### Data collection

**Figure 2:**
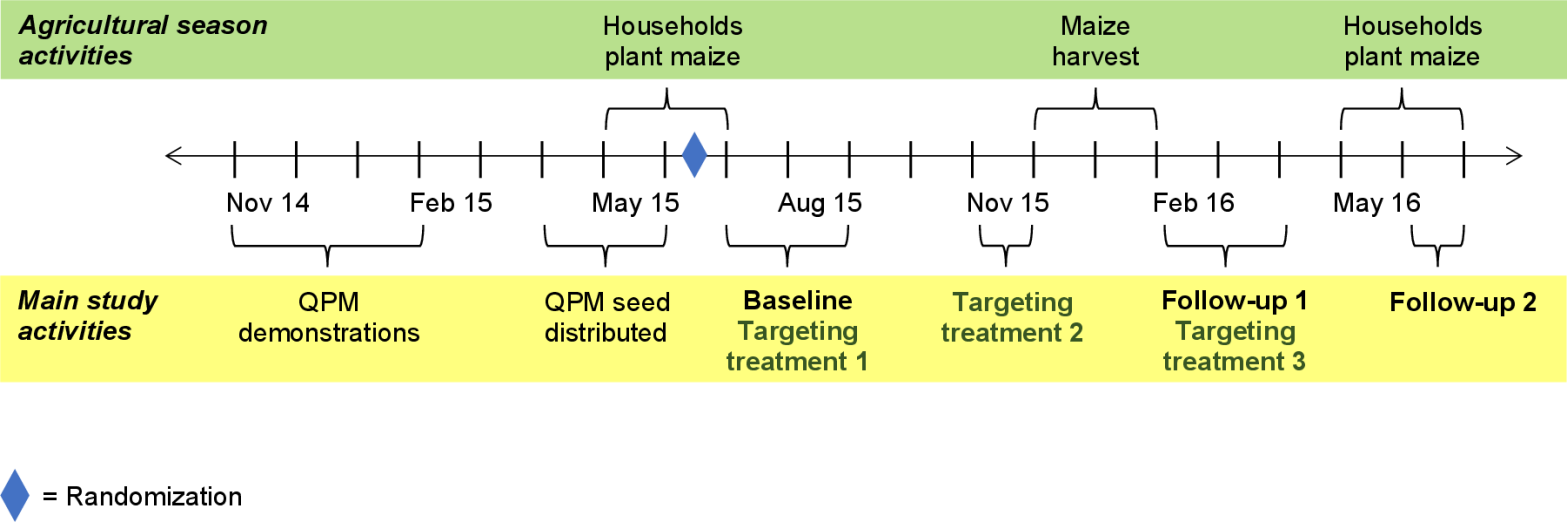
Time line. Targeting treatment 1: information about nutritional benefits of QPM and importance of keeping QPM separate during harvest and storage provided to household head, information about nutritional benefits and importance of targeting QPM provided to caregiver; targeting treatment 2: group meetings with caregivers to discuss child nutrition and targeting and distribute labelled flour and grain bags, bowl and spoon; targeting treatment 3: refresher on nutritional benefits of QPM and importance of targeting provided to the caregiver just after the follow-up 1 survey. QPM, quality protein maize.

A baseline survey was administered after maize planting (figure 2). Two follow-up visits were conducted 1–3 months (follow-up 1) and 5–6 months (follow-up 2) after the maize harvest. All components of the targeting intervention happened before follow-up 1, except for a refresher on the main intervention messages that was provided after the conclusion of administering the survey instrument at the time of follow-up 1. Participants’ flow through the study is presented in online supplemental figure A1.

10.1136/bmjgh-2020-002705.supp1 Supplementary data

The baseline survey included household demographics, information on cooking and feeding practices and anthropometric measures including height and weight. Key survey modules for the follow-up surveys included 7-day Food-Frequency Questionnaires, caregiver cooking and feeding behaviours and household maize storage practices. Anthropometric measurements were collected for the index child at each follow-up. Additional details regarding data collection are available in other work.^36 37^

### Outcomes

Study outcomes capture the impact on agricultural, cooking and feeding practices targeted by the intervention, including indicators measured at the household level for whether: (1) QPM grain was stored separately from other maize grain, (2) QPM flour was stored separately from other maize flour, (3) QPM was not sold, (4) the caregiver cooked food with QPM specifically for children under 5 in the last 7 days, (5) the index child consumed QPM in the last 7 days, (6) the index child consumed porridge made from QPM in the last 7 days, (7) the index child ate QPM for more days than the household head (indicator only available during follow-up 1 survey) and (8) the index child ate from his/her own plate. Outcomes related to the storage of QPM were observed by the enumerator, while all other outcomes were self-reported. A summary ‘compliance index’ based on the share of these targeted behaviours (out of 8) the household reported performing was constructed. This compliance index takes on up to nine different values varying between 0 and 1. In order to explore the extent of the behaviour change with respect to cooking and feeding, we also report the number of days that (1) the caregiver cooked a food with QPM specifically for young children under 5, (2) the index child consumed any QPM in the last 7 days and (3) the index child consumed porridge made with QPM in the last 7 days.

All outcomes measuring QPM-related practices were preregistered, though in some cases the formulation of our variable differs slightly from what is specified in the registry. For example, we preregistered an intention to analyse an outcome related to whether the caregiver cooked for the index child, however, as the survey instrument asked about all children under 5 we modified the outcome definition accordingly. The compliance outcome was not a preregistered study outcome, but provides a summary measure across behavioural outcomes. Analysing an outcome index allows us to summarise the effects of the intervention on targeted outcomes while minimising the potential challenges that arise from multiple hypothesis testing^40^ due to the large number of behavioural outcomes of interest.

We also report anthropometric outcomes, including height-for-age and weight-for-age z scores (based on 2006 WHO growth standards) for the index child. In the main text, we report anthropometric outcomes at both follow-up 1 and follow-up 2 and focus on behavioural change measures at follow-up 1 only, which took place after the harvest but before households would have run out of QPM. The outcomes measured at follow-up 1 provide evidence about the intervention impacts while messaging was still recent and during a time when households were less likely to have been experiencing food insecurity. In the online supplemental appendix, we report the intraclass correlation coefficient for each primary outcome (online supplemental table A1); we also present behavioural change measures at follow-up 2 (online supplemental table A3) and evidence about whether the intervention led to changes in consumption of other protein rich foods such as meat, eggs, legumes or milk (online supplemental table A4).

### Statistical analysis

Baseline characteristics of the sample by treatment arm are presented, with p values from t-tests of the difference between treatment arms, as well as the F-statistic from a joint test of orthogonality. We estimated intention-to-treat effects of the targeting intervention using ordinary least squares regression models for ease of interpretation.^41^ Alternative models with logistic regression for binary outcomes and Poisson regression for number of days are included in online supplemental tables A5–A7). Each outcome was regressed on a dummy variable representing assignment to the ‘QPM+targeting’ group as well as kebele fixed effects to account for stratification. All analyses included heteroscedasticity robust standard errors clustered at the community health group level (the level of randomisation) to account for clustering.^42^ We report regression estimates adjusted only for stratification variables (partially adjusted) and adjusted for all baseline household, caregiver and index child characteristics included in table 1 (fully adjusted) following guidance from Bruhn and McKenzie to report a full set of adjusted covariates instead of only those experiencing imbalance.^43^ Because of chance imbalance in baseline covariates, we report estimates from the fully adjusted model as our primary model.

**Table 1:** Baseline characteristics of the study participants (final sample)

	Total	QPM only	QPM+targeting	P value
	Mean (SD)	Mean (SD)	Mean (SD)	
Household characteristics				
No of household members*	6.2 (2.0)	6.2 (2.0)	6.2 (2.1)	0.99
High quality roof*†	57.3% (49.5%)	60.7% (48.9%)	53.3% (50.0%)	0.09
Land owned (timad)*†	5.6 (6.3)	5.6 (6.6)	5.5 (6.0)	0.64
Mazie produced (kg)*†	1869.4 (2391.0)	1719.3 (2342.6)	2042.4 (2438.6)	0.45
Caregiver characteristics				
Age (years)*	28.5 (5.8)	28.5 (5.7)	28.4 (6.0)	0.82
Attended school*	33.3% (47.2%)	29.6% (45.7%)	37.6% (48.5%)	0.07
No of pregnancies*	4.4 (2.3)	4.5 (2.1)	4.4 (2.4)	0.79
Index child characteristics				
Age (months)*	19.9 (8.4)	19.1 (7.9)	20.8 (8.9)	0.02
Male*	51.9% (50.0%)	55.9% (49.7%)	47.4% (50.0%)	0.06
Height-for-age (z-score)*	−1.4 (1.4)	−1.5 (1.4)	−1.2 (1.5)	0.04
Weight-for-age (z-score)*	−1.0 (1.1)	−1.1 (1.1)	−0.9 (1.2)	0.12
Health and health seeking behaviour				
Index child sick with diarrhoea in past 2 weeks	17.4% (37.9%)	19.1% (39.4%)	15.4% (36.2%)	0.32
Index child sick with fever in past 2 weeks	18.9% (39.2%)	20.1% (40.1%)	17.7% (38.2%)	0.29
No times caregiver sought antenatal care during pregnancy with index child	3.2 (1.6)	3.2 (1.7)	3.2 (1.5)	0.93
Cooking and feeding				
Days in past week cooked specifically for young children	1.8 (2.3)	1.8 (2.2)	1.8 (2.3)	0.94
Days in past week cooked something with maize	5.7 (2.5)	5.7 (2.5)	5.7 (2.4)	0.98
Days in past week index child ate food with QPM	0.1 (0.8)	0.1 (0.7)	0.2 (0.9)	0.57
Days in past week index child ate porridge	0.8 (1.2)	0.8 (1.2)	0.8 (1.2)	0.43
Worried not enough food because not enough money, in last 3 months	37.5% (48.5%)	39.8% (49.0%)	35.0% (47.8%)	0.21
Attrition		5.0% (21.8%)	8.3% (27.6%)	0.16
Joint test of orthogonality F-statistic			1.9	0.01
N households	570	304	266	
N clusters	369	195	174	

The final sample is defined as all households where the caregiver survey was conducted at baseline, follow-up 1 and follow-up 2. P values are derived from a regression of the outcome on an indicator for the QPM+targeting group, controlling for kebele (strata) and clustered at the community health group level. Child height-for-age and weight-for-age z-scores are normalised using the 2006 WHO growth standards. The joint test of orthogonality is a test of the null hypothesis that the coefficients on all characteristics in the table are jointly equal to 0, where the outcome is an indicator for the QPM+targeting group.

*Baseline characteristics included in the regression as covariates.

†Rows are measured through the household head survey, QPM only n=303, QPM+targeting n=261.

QPM, quality protein maize.

### Statistical power

The study was powered to detect changes in anthropometric and biomarker outcomes that will be primarily analysed in other work.^36^ We report the ex-post minimum detectable effect sizes at 80% power for cooking, feeding and agricultural practices using the SEs estimated from the partially adjusted model to demonstrate our statistical power based on the realised data (online supplemental table A8).

### Patient and public involvement

The public were not involved in the design, implementation, reporting or dissemination of this research.

## Results

The sample included 610 eligible households, with 320 households in the QPM-only group and 290 in the QPM+targeting group. Sixteen households in the QPM-only group and 24 in the QPM+targeting group were lost to follow-up, leaving an analytical sample of 304 households in the QPM-only group and 266 households in the QPM+targeting group (online supplemental figure A1). Baseline characteristics of the analytical sample for whom we can observe outcomes are reported in table 1. The average household in the sample had six members and owned 5.6 timads (roughly 1.4 hectare) of agricultural land. The average index child was 20 months old with height-for-age and weight-for-age z scores at −1.4 and −1.0 SD, respectively. Households reported minimal consumption of QPM in the past week. Household and caregiver characteristics were similar across treatment arms but children in the QPM+ targeting arm are slightly older (20.8 months vs 19.1 months; p=0.02) with higher height-for-age z scores (−1.2 vs −1.5; p=0.04). Descriptive statistics are similar when estimated for the sample including all households surveyed at baseline including those eventually lost to follow-up (online supplemental table A2).

Households in the QPM-only group performed 41.3% of the targeted storage, cooking and feeding behaviours in the compliance index (table 2). The targeting intervention increased the compliance index by 20.5 percentage points (pp) (95% CI 16.7 pp to 24.4 pp; p<0.01), which is equivalent to performing 1.6 more targeted behaviours. At follow-up 2, households in the QPM-only group performed 42.9% of the targeted behaviours, similar to their mean level at follow-up 1 (online supplemental table A3). For the QPM+ targeting group, behavioural change was attenuated at follow-up 2, but households in this arm still performed 9.7 pp (95% CI 5.0 pp to 14.3 pp; p<0.01) more of the targeted behaviours.

**Table 2:** Impact of the intervention package on behavioural outcomes at follow-up 1 (OLS)

	Mean at follow-up 1	Partially adjusted		Adjusted	
		Beta (95% CI)	P value	Beta (95% CI)	P value
**Overall compliance**					
Percentage share of targeted behaviours performed					
QPM only	41.3%				
QPM+targeting	62.6%	21.8 pp (18.2 pp to 25.4 pp)	<0.01	20.5 pp (16.7 pp to 24.4 pp)	<0.01
**Panel A: storing and selling QPM**					
QPM grain unmixed during storage*†					
QPM only	39.8%				
QPM+ targeting	83.1%	42.8 pp (35.3 pp to 50.2 pp)	<0.01	39.7 pp (32.0 pp to 47.5 pp)	<0.01
QPM flour unmixed during storage†					
QPM only	26.0%				
QPM+targeting	74.1%	48.0 pp (40.2 pp to 55.8 pp)	<0.01	46.5 pp (38.3 pp to 54.7 pp)	<0.01
Not sold QPM since beginning of season†					
QPM only	92.1%				
QPM+ targeting	94.7%	4.3 pp (0.5 pp to 8.1 pp)	0.03	4.9 pp (0.7 pp to 9.0 pp)	0.02
**Panel B: cooking**					
Cooked QPM food specifically for young children†					
QPM only	9.9%				
QPM+targeting	24.8%	15.0 pp (9.0 pp to 21.0 pp)	<0.01	14.4 pp (7.9 pp to 20.9 pp)	<0.01
Days cooked QPM food specifically for young children					
QPM only	0.2				
QPM+targeting	0.8	0.6 (0.4 to 0.8)	<0.01	0.6 (0.4 to 0.8)	<0.01
**Panel C: feeding and consumption**					
Index child consumed any QPM in last 7 days†					
QPM only	62.8%				
QPM+targeting	81.6%	19.3 pp (12.2 pp to 26.4 pp)	<0.01	17.3 pp (9.4 pp to 25.1 pp)	<0.01
Days index child consumed QPM last week					
QPM only	3.6				
QPM+targeting	4.5	1.0 (0.5 to 1.4)	<0.01	0.9 (0.4 to 1.4)	<0.01
Index child consumed porridge with QPM last week†					
QPM only	31.6%				
QPM+ targeting	55.6%	24.8 pp (17.1 pp to 32.5 pp)	<0.01	22.0 pp (13.6 pp to 30.4 pp)	<0.01
Days index child consumed porridge with QPM last week					
QPM only	0.6				
QPM+targeting	1.2	0.5 (0.4 to 0.7)	<0.01	0.4 (0.3 to 0.6)	<0.01
Index child ate QPM for more days than household head†					
QPM only	3.9%				
QPM+targeting	17.3%	13.0 pp (8.0 pp to 18.1 pp)	<0.01	12.2 pp (6.8 pp to 17.6 pp)	<0.01
Difference in no of days QPM consumed between index child and household head					
QPM only	0.0				
QPM+targeting	0.6	0.6 (0.4 to 0.8)	<0.01	0.5 (0.3 to 0.7)	<0.01
Index child ate from own plate†					
QPM only	64.5%				
QPM+targeting	69.9%	7.1 pp (−0.8 pp to 15.1 pp)	0.08	7.4 pp (−1.1 pp to 15.9 pp)	0.09

*Questions refer to how QPM was previously stored if household had already run out of QPM at the time of the first follow-up survey. Coefficients from ordinary least squares models are reported. Partially adjusted models only control for kebele to account for stratification and are clustered at the community health group level; adjusted models additionally control for household, caregiver and index child characteristics shown in table 1.

†Only items marked with '†' are in the overall compliance measure, which include (1) stored QPM grain separately, (2) stored QPM flour separately, (3) did not sell QPM, (4) cooked QPM specifically for young children, (5) index child ate QPM last week, (6) index child consumed QPM porridge last week, (7) index child ate QPM for more days than head of household, and (8) index child ate from own plate.

Beta, linear regression coefficient; PP, percentage points; QPM, Quality Protein Maize.

Households in the QPM+ targeting arm were 39.7 pp (95% CI 32.0 pp to 47.5 pp; p<0.01) and 46.5 pp (95% CI 38.3 pp to 54.7 pp; p<0.01) more likely to keep QPM grain unmixed and QPM flour unmixed, respectively, during storage compared with the QPM-only group (table 2). They were 14.4 pp more likely to cook foods with QPM specifically for their young children (95% CI 7.9 pp to 20.9 pp; p<0.01), on average cooking foods with QPM 0.6 more days in the past week (95% CI 0.4 to 0.8; p<0.01) than the QPM-only group. Relative to the QPM-only group—where 62.8% of index children consumed QPM in the last week—index children in the QPM+targeting group were 17.3 pp (95% CI 9.4 pp to 25.1 pp; p<0.01) more likely to have consumed QPM in the past week, consuming QPM an average of 0.9 more days during the week (95% CI 0.4 to 1.4 days; p<0.01). Index children in the QPM+targeting arm were also 22.0 pp (95% CI 13.6 pp to 30.4 pp; p<0.01) more likely to consume porridge made with QPM in the last week and 12.2 pp (95% CI 6.8 pp to 17.6 pp; p<0.01) more likely to have consumed more QPM than the household head in the last week than the QPM-only group. No significant difference across arms was found in the probability that the index child ate from his/her own plate in the most recent meal, though the estimated effect is positive and practically meaningful (7.4 pp; 95% CI −1.1 pp to 15.9 pp; p=0.09). Similar results are found for alternative model specifications (online supplemental tables A5–A6). We found suggestive evidence for substitution from conventional maize to QPM in the QPM+targeting group but limited evidence of substitution away from other protein-rich foods groups (online supplemental table A4 and A7).

No statistically significant differences in anthropometric outcomes between the QPM-only and the QPM+targeting arms were found at either follow-up point (table 3).

**Table 3:** Impact of the intervention package on anthropometrics outcomes (OLS)

		Partially adjusted		Adjusted	
	Mean	Beta (95% CI)	P value	Beta (95% CI)	P value
**Panel A: follow-up 1**					
HAZ					
QPM only	−1.5				
QPM+targeting	−1.3	0.2 (−0.1 to 0.4)	0.15	−0.1 (−0.2 to 0.0)	0.07
WAZ					
QPM only	−1.2				
QPM+targeting	−1.0	0.1 (−0.1 to 0.3)	0.23	−0.0 (−0.1 to 0.1)	0.36
**Panel B: follow-up 2**					
HAZ					
QPM only	−1.7				
QPM+targeting	−1.5	0.2 (−0.0 to 0.4)	0.08	−0.0 (−0.1 to 0.1)	0.43
WAZ					
QPM only	−1.1				
QPM+targeting	−1.0	0.1 (−0.1 to 0.2)	0.53	−0.1 (−0.2 to 0.1)	0.30

Child height-for-age (HAZ) and weight-for-age (WAZ) z-scores are normalised using the 2006 WHO growth standards. Coefficients from ordinary least squares models are reported. Partially adjusted models only control for kebele to account for stratification and are clustered at the community health group level; adjusted models additionally control for household, caregiver and index child characteristics shown in table 1.

Beta, linear regression coefficient; QPM, quality protein maize.

## Discussion

Results from this study highlight the potential challenges to improving child dietary quality via the dissemination of biofortified crop varieties. Translating adoption of biofortified varieties to child consumption requires simultaneous changes to agricultural, cooking and feeding practices, which can be time-consuming and unfamiliar for caregivers.^44^ We find that, among households that only received QPM seed, more than one-third of children did not consume any QPM over the course of a week. Further, even for those children who were consuming QPM, the benefits were likely highly diluted, as three-quarters of households had mixed the QPM flour with conventional maize. This evidence is consistent with other studies that have documented limited adoption and challenges with compliance when biofortified or fortified food has been introduced in uncontrolled settings.^13 24 25^

Our results suggest that it is possible to elicit changes in behaviours that can help translate adoption of biofortified crop varieties into child consumption. The targeting interventions tested here—which combined light-touch behavioural interventions with heavier-touch efforts such as separate storage and feeding containers—led to changes in a variety of targeted behaviours, including the consumption of QPM for young children. The impacts were substantial: the intervention increased QPM consumption for young children by nearly 25% and more than doubled the probability that the caregiver cooked a QPM food specifically for young children. While behavioural changes attenuated over time—possibly due in part to the fact that the second follow-up was collected when roughly a third of households had no remaining QPM—statistically significant changes in behaviours persisted. Prior studies have documented that the impact of interventions on nutritional outcomes varies significantly with compliance,^45^ and that feeding and cooking practices are particularly difficult to change.^46 47^ Our study provides evidence that interventions informed by behavioural insights that consider a variety of barriers to consumption may enable the translation of biofortified crop varieties into meaningful improvements in consumption for young children.

There are several reasons that increased consumption of QPM may not have translated into significant improvements in growth. It could be that the increase in QPM consumption was not enough to lead to meaningful changes in growth,^48^ or that the amount of time children had been eating QPM was not long enough to detect impacts. It is also possible that impurities in the QPM grain (resulting from cross pollination with conventional maize, which can occur on small plots) resulted in less available quality protein. Finally, imbalance in some baseline anthropometric characteristics, despite randomisation, meant that estimates of anthropometric impacts were sensitive to the inclusion of these baseline measures. Future research should continue to investigate the relationship between consumption of QPM in uncontrolled setting and changes in child growth.

Our study has several limitations. First, our indicators of behavioural change rely largely on self-report. Given that our data collection team also delivered key elements of the intervention, families in the treatment arm may have been more likely to report desired behaviours. Nonetheless, positive impacts were found for outcomes that were verified through observation, for example, storing QPM separately from other grains and on outcomes measured by the data collection team prior to delivering study interventions. Second, baseline imbalance in anthropometrics makes it difficult to draw firm conclusions about the impact of the intervention on growth outcomes. Nonetheless, behavioural outcomes were robust to inclusion of these baseline covariates. Finally, because our intervention sought to alter a number of relevant behaviours along the causal pathway toward increased consumption of QPM by small children, we preregistered a large number of study outcomes. We attempt to mitigate the challenges of examining a large number of outcomes by summarising impacts in a post hoc behavioural ‘compliance index,’ similar to approaches taken in previous social science trials.^40^

While governments and international agencies have increasingly recognised the importance of improving child nutrition, research has largely focused on nutrient supplementation,^49^ with less evidence on the effectiveness of interventions to improve diet quality.^50^ Our results suggest that behavioural tools and messaging, which have been applied to many global health challenges but have been limited in their application to global nutrition,^51^ may represent effective levers for changing feeding practices to improve child dietary quality. The Ethiopian government has set the goal of converting 10% of the country’s maize production to QPM within a few years^52^; while this is an important first step, this study shows that additional, complementary policy initiatives may be needed in order to achieve the ultimate goal of biofortified crops: improved nutrition.
