# Supplementary material for: Behavioural insights to support increased consumption of quality protein maize by young children: a cluster randomised trial in Ethiopia
Source: BMJ Glob Health. 2020 Dec 18;5(12):e002705. doi: 10.1136/bmjgh-2020-002705 (PMC7751204; doi:10.1136/bmjgh-2020-002705)
Supplement: Supplementary data [file bmjgh-2020-002705supp001.pdf]

Figure A1. Full Consort Diagram

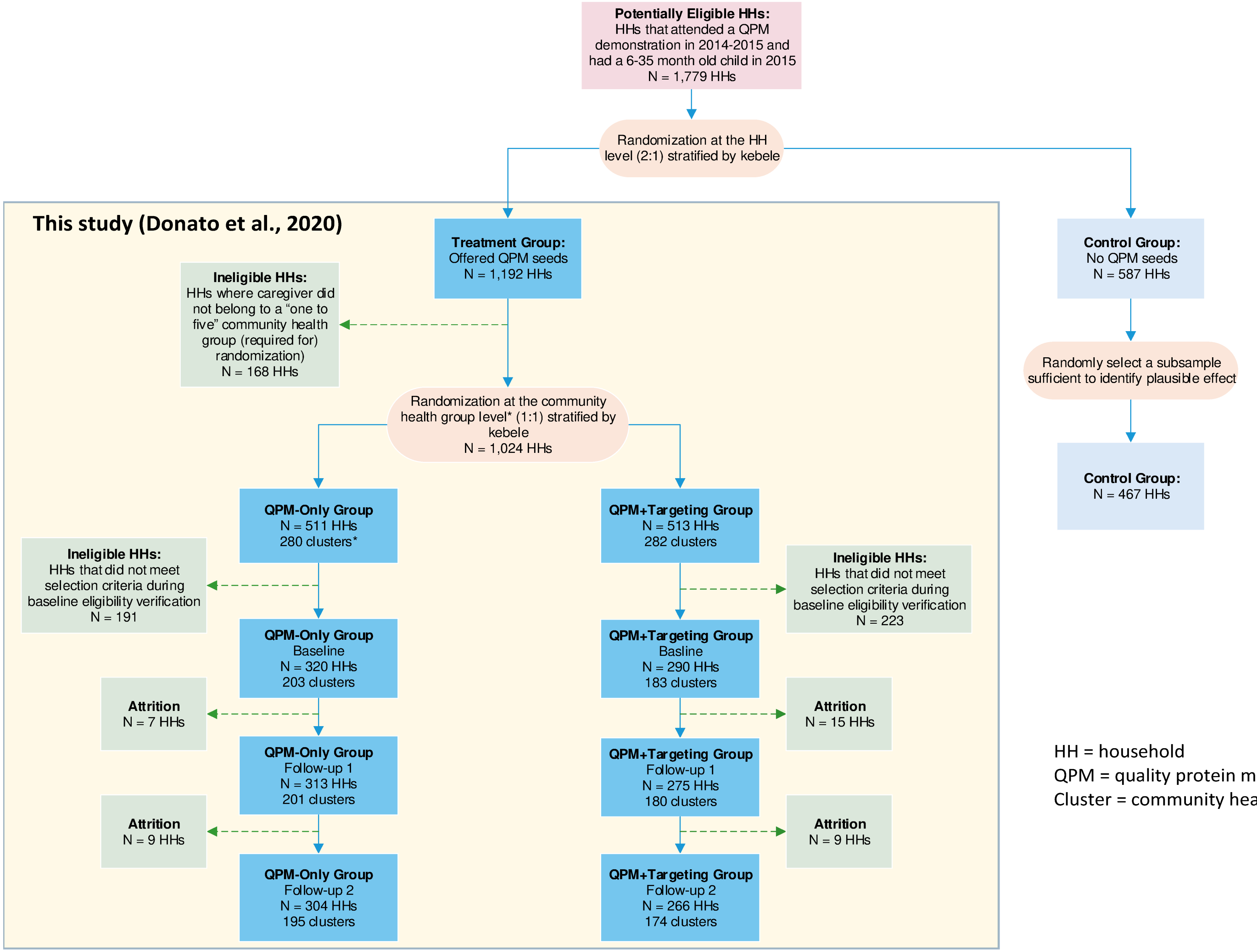

HH = household
QPM = quality protein maize
Cluster = community health group

Table A1. Intraclass correlation for primary outcomes

**Primary outcomes (follow-up 1 measures)**	**Intraclass correlation**
**Overall compliance**	
Percentage share of targeted behaviors performed	0.297
**Storing and selling QPM**	
QPM grain unmixed during storage	0.346
QPM flour unmixed during storage	0.350
Not sold QPM since beginning of season	0.200
**Cooking**	
Cooked QPM food specificially for young children	0.209
Days cooked QPM food specifically for young children	0.505
**Feeding**	
Index child consumed any QPM in last 7 days	0.100
Days index child consumed QPM last week	0.156
Index child consumed porridge with QPM last week	0.152
Days index child consumed porridge with QPM last week	0.224
Index child ate QPM for more days than household head	0.425
Difference in number of days QPM consumed between index child and household head	0.357
Index child ate from own plate	0.112
**Index child anthropometrics**	
Index child height-for-age	0.173
Index child weight-for-age	0.177

Notes: QPM = quality protein maize.

Table A2. Baseline characteristics of the study participants (baseline sample)

	Total	QPM only	QPM + targeting	
	**Mean (SD)**	**Mean (SD)**	**Mean (SD)**	**p-value**
Household Characteristics				
Number of household members^	6.2 (2.1)	6.2 (2.0)	6.2 (2.2)	0.80
High quality roof*^	56.4% (49.6%)	59.7% (49.1%)	52.6% (50.0%)	0.07
Land owned (timad)*^	5.5 (6.3)	5.6 (6.6)	5.4 (5.9)	0.67
Mazie produced(Kg)*^	1881.9 (2362.5)	1744.2 (2336.6)	2034.4 (2385.7)	0.60
Caregiver Characteristics				
Age (years)^	28.4 (5.8)	28.4 (5.7)	28.3 (6.0)	0.97
Attended school^	34.6% (47.6%)	30.3% (46.0%)	39.3% (48.9%)	0.04
Number of pregnancies^	4.4 (2.3)	4.5 (2.2)	4.3 (2.4)	0.86
Index Child Characteristics				
Age (months)^	20.0 (8.4)	19.0 (7.9)	21.2 (8.9)	<0.01
Male^	51.6% (50.0%)	55.6% (49.8%)	47.2% (50.0%)	0.06
Height-for-age (z-score)^	-1.4 (1.4)	-1.5 (1.3)	-1.2 (1.5)	0.07
Weight-for-age (z-score)^	-1.0 (1.1)	-1.1 (1.1)	-0.9 (1.1)	0.15
Health and Health-Seeking Behavior				
Index child sick with diarrhea in past 2 weeks	18.0% (38.5%)	19.1% (39.3%)	16.9% (37.5%)	0.50
Index child sick with fever in past 2 weeks	19.3% (39.5%)	20.9% (40.7%)	17.6% (38.1%)	0.17
Number times caregiver sought ANC during pregnancy with index child	3.2 (1.6)	3.2 (1.6)	3.3 (1.5)	0.75
Cooking and Feeding				
Days in past week cooked specifically for young children	1.8 (2.3)	1.8 (2.3)	1.8 (2.3)	0.85
Days in past week cooked something with maize	5.7 (2.5)	5.6 (2.5)	5.7 (2.4)	0.99
Days in past week index child ate food with QPM	0.1 (0.8)	0.1 (0.7)	0.2 (0.8)	0.78
Days in past week index child ate porridge	0.8 (1.2)	0.8 (1.2)	0.8 (1.2)	0.47
Worried not enough food because not enough money, in last 3 mos.	37.2% (48.4%)	38.8% (48.8%)	35.5% (47.9%)	0.43
Joint test of orthogonality F-statistic			2.1	0.01
N households	610	320	290	
N clusters	386	203	183	

Notes: The baseline sample is defined as all households where the caregiver survey was conducted at baseline. SD = standard deviation. P-values are derived from a regression of the outcome on an indicator for the QPM + targeting group, controlling for kebele (strata) and clustered at the community health group level. Child height-for-age and weight-for age z-scores are normalized using the 2006 WHO growth standards. The joint test of orthogonality is a test of the null hypothesis that the coefficients on all characteristics in the table are jointly equal to zero, where the outcome is an indicator for the QPM + targeting group. *Rows are measured through the household head survey, QPM only N = 318, QPM + targeting N = 285. ^Baseline characteristics included in the regression as covariates.

Table A3. Impact of the intervention package on behavioral outcomes at follow-up 2 (OLS)

	Mean at follow-up 2	Partially adjusted		Adjusted	
		Beta (95% CI)	p-value	Beta (95% CI)	p-value
Overall Compliance					
Percentage share of targeted behaviors performed					
QPM only	42.9%				
QPM + targeting	54.0%	11.7 pp (7.3 pp to 16.1 pp)	<0.01	9.7 pp (5.0 pp to 14.3 pp)	<0.01
**Panel A: Storing and Selling QPM**					
QPM grain unmixed during storage‡					
QPM only	74.1%				
QPM + targeting	94.8%	20.7 pp (13.0 pp to 28.4 pp)	<0.01	22.2 pp (13.8 pp to 30.6 pp)	<0.01
QPM flour unmixed during storage‡					
QPM only	59.3%				
QPM + targeting	87.4%	28.0 pp (19.0 pp to 37.0 pp)	<0.01	30.1 pp (20.7 pp to 39.4 pp)	<0.01
Never sold QPM‡					
QPM only	88.2%				
QPM + targeting	91.4%	5.1 pp (0.2 pp to 10.0 pp)	0.04	5.2 pp (-0.4 pp to 10.8 pp)	0.07
**Panel B: Cooking**					
Cooked QPM food specifically for young children‡					
QPM only	11.5%				
QPM + targeting	17.7%	6.6 pp (0.9 pp to 12.4 pp)	0.02	4.5 pp (-1.5 pp to 10.4 pp)	0.14
Days cooked QPM food specifically for young children					
QPM only	0.3				
QPM + targeting	0.5	0.2 (0.0 to 0.4)	0.02	0.2 (-0.0 to 0.4)	0.06
**Panel C: Feeding and Consumption**					
Index child consumed any QPM in last 7 days‡					
QPM only	50.7%				
QPM + targeting	58.3%	7.8 pp (-0.8 pp to 16.3 pp)	0.07	3.2 pp (-5.7 pp to 12.2 pp)	0.48
Days index child consumed QPM last week					
QPM only	2.9				
QPM + targeting	3.2	0.3 (-0.2 to 0.8)	0.28	0.1 (-0.5 to 0.6)	0.76
Index child consumed porridge with QPM last week‡					
QPM only	26.6%				
QPM + targeting	35.0%	8.8 pp (1.1 pp to 16.5 pp)	0.03	5.8 pp (-2.2 pp to 13.8 pp)	0.16
Days index child consumed porridge with QPM last week					
QPM only	0.5				
QPM + targeting	0.6	0.2 (0.0 to 0.3)	0.02	0.1 (-0.0 to 0.3)	0.10
Index child ate from own plate‡					
QPM only	52.3%				
QPM + targeting	56.4%	5.7 pp (-2.8 pp to 14.2 pp)	0.19	4.7 pp (-4.1 pp to 13.6 pp)	0.29

Notes: Beta = linear regression coefficient; CI = confidence interval; QPM = Quality Protein Maize; pp = percentage points *Questions are only asked in cases where the household had QPM remaining at follow-up 2. Coefficients from ordinary least squares models are reported. Partially adjusted models only control for kebele to account for stratification and are clustered at the community health group level; adjusted models additionally control for household, caregiver and index child characteristics shown in Table 1. ‡Only items marked with '‡' are in the overall compliance measure, which include 1) stored QPM flour separately, 2) stored QPM grain separately, 3) did not sell QPM, 4) cooked QPM specifically for young children, 5) index child consumed QPM porridge last week, 6) index child ate from own plate, and 7) index child ate QPM last week.

Table A4. Impact of the intervention package on food group consumption (OLS)

		Partially adjusted		Adjusted	
	**Mean**	**Beta (95% CI)**	**p-value**	**Beta (95% CI)**	**p-value**
**Panel A: Follow-up 1**					
Number of days index child consumed conventional maize in last week					
QPM only	4.6				
QPM + targeting	4.2	-0.5 (-0.9 to 0.0)	0.07	-0.4 (-0.9 to 0.2)	0.17
Number of days index child consumed QPM in last week					
QPM only	3.6				
QPM + targeting	4.5	1.0 (0.5 to 1.4)	<0.01	0.9 (0.4 to 1.4)	<0.01
Number of days index child consumed meat or fish in last week					
QPM only	0.5				
QPM + targeting	0.5	-0.0 (-0.2 to 0.1)	0.75	-0.0 (-0.2 to 0.2)	0.80
Number of days index child consumed eggs in last week					
QPM only	0.7				
QPM + targeting	0.6	-0.0 (-0.2 to 0.2)	0.74	-0.1 (-0.3 to 0.1)	0.52
Number of days index child consumed legumes in last week					
QPM only	4.5				
QPM + targeting	4.5	-0.1 (-0.5 to 0.3)	0.66	-0.1 (-0.4 to 0.3)	0.79
Number of days index child consumed milk in last week					
QPM only	1.8				
QPM + targeting	1.8	0.1 (-0.4 to 0.5)	0.74	-0.1 (-0.5 to 0.4)	0.73
**Panel B: Follow-up 2**					
Number of days index child consumed conventional maize in last week					
QPM only	5.1				
QPM + targeting	4.9	-0.3 (-0.7 to 0.2)	0.24	-0.1 (-0.6 to 0.4)	0.75
Number of days index child consumed QPM in last week					
QPM only	2.9				
QPM + targeting	3.2	0.3 (-0.2 to 0.8)	0.28	0.1 (-0.5 to 0.6)	0.76
Number of days index child consumed meat or fish in last week					
QPM only	0.5				
QPM + targeting	0.5	0.1 (-0.1 to 0.3)	0.26	0.1 (-0.1 to 0.3)	0.34
Number of days index child consumed eggs in last week					
QPM only	0.7				
QPM + targeting	0.6	0.0 (-0.2 to 0.2)	0.96	-0.0 (-0.2 to 0.2)	0.64
Number of days index child consumed legumes in last week					
QPM only	4.8				
QPM + targeting	4.9	0.0 (-0.3 to 0.4)	0.83	0.1 (-0.3 to 0.5)	0.53
Number of days index child consumed milk in last week					
QPM only	2.2				
QPM + targeting	2.2	0.0 (-0.4 to 0.5)	0.84	-0.1 (-0.6 to 0.4)	0.74

Notes: Beta = linear regression coefficient; CI = confidence interval. Coefficients from ordinary least squares models are reported. Partially adjusted models only control for kebele to account for stratification and are clustered at the community health group level; adjusted models additionally control for household, caregiver and index child characteristics shown in Table 1.

Table A5. Logit and Poisson regressions of impact of the intervention package on behavioral outcomes at follow-up 1

		Partially adjusted		Adjusted	
	**Mean at follow-up 1**	**OR or IRR (95% CI)**	**p-value**	**OR or IRR (95% CI)**	**p-value**
**Panel A: Storing and Selling QPM**					
QPM grain unmixed during storage*‡					
QPM only	39.8%				
QPM + targeting	83.1%	8.9 (5.8 to 13.7)	<0.01	8.5 (5.3 to 13.6)	<0.01
QPM flour unmixed during storage*‡					
QPM only	26.0%				
QPM + targeting	74.1%	8.7 (5.7 to 13.3)	<0.01	8.5 (5.5 to 13.3)	<0.01
Not sold QPM since beginning of season‡					
QPM only	92.1%				
QPM + targeting	94.7%	2.3 (1.1 to 5.2)	0.04	2.7 (1.2 to 6.5)	0.02
**Panel B: Cooking**					
Cooked QPM food specifically for young children‡					
QPM only	9.9%				
QPM + targeting	24.8%	3.4 (2.0 to 5.6)	<0.01	3.2 (1.8 to 5.6)	<0.01
Days cooked QPM food specifically for young children					
QPM only	0.2				
QPM + targeting	0.8	3.9 (2.4 to 6.2)	<0.01	3.6 (2.2 to 6.0)	<0.01
**Panel C: Feeding and Consumption**					
Index child consumed any QPM in last 7 days‡					
QPM only	62.8%				
QPM + targeting	81.6%	3.0 (1.9 to 4.5)	<0.01	2.8 (1.7 to 4.4)	<0.01
Days index child consumed QPM last week					
QPM only	3.6				
QPM + targeting	4.5	1.3 (1.1 to 1.4)	<0.01	1.2 (1.1 to 1.4)	<0.01
Index child consumed porridge with QPM last week‡					
QPM only	31.6%				
QPM + targeting	55.6%	3.0 (2.1 to 4.4)	<0.01	2.8 (1.9 to 4.1)	<0.01
Days index child consumed porridge with QPM last week					
QPM only	0.6				
QPM + targeting	1.2	1.9 (1.5 to 2.3)	<0.01	1.7 (1.4 to 2.2)	<0.01
Index child ate QPM for more days than household head‡					
QPM only	3.9%				
QPM + targeting	17.3%	5.8 (2.9 to 11.6)	<0.01	5.6 (2.6 to 12.0)	<0.01
Difference in number of days QPM consumed between index child and household head**					
QPM only	0.0				
QPM + targeting	0.6	0.6 (0.4 to 0.8)	<0.01	0.5 (0.3 to 0.7)	<0.01
Index child ate from own plate‡					
QPM only	64.5%				
QPM + targeting	69.9%	1.4 (1.0 to 2.1)	0.08	1.5 (1.0 to 2.3)	0.07

Notes: OR = odds ratio for indicator outcomes from logistic regression; IRR = incidence rate ratio for days outcomes from Poisson regression; CI = confidence interval; QPM = Quality Protein Maize. *Questions refer to how QPM was previously stored if household had already run out of QPM at the time of the 1st follow-up survey. Coefficients from ordinary least squares models are reported. Partially adjusted models only control for kebele to account for stratification and are clustered at the community health group level; adjusted models additionally control for household, caregiver and index child characteristics shown in Table 1. **Variable has negative values so results from OLS model are reported instead. ‡Only items marked with '‡' are in the overall compliance measure; the effect on overall compliance is estimated using OLS only and therefore excluded from this table.

Table A6. Logit and Poisson regressions of impact of the intervention package on behavioral outcomes at follow-up 2

		Partially adjusted		Adjusted	
	**Mean at follow-up 2**	**OR or IRR (95% CI)**	**p-value**	**OR or IRR (95% CI)**	**p-value**
**Panel A: Storing and Selling QPM**					
QPM grain unmixed during storage‡					
QPM only	74.1%				
QPM + targeting	94.8%	6.8 (2.9 to 16.1)	<0.01	9.1 (3.1 to 26.8)	<0.01
QPM flour unmixed during storage‡					
QPM only	59.3%				
QPM + targeting	87.4%	5.2 (2.9 to 9.4)	<0.01	6.5 (3.4 to 12.6)	<0.01
Never sold QPM‡					
QPM only	88.2%				
QPM + targeting	91.4%	1.9 (1.0 to 3.6)	0.05	1.9 (1.0 to 3.9)	0.06
**Panel B: Cooking**					
Cooked QPM food specifically for young children‡					
QPM only	11.5%				
QPM + targeting	17.7%	1.8 (1.1 to 2.9)	0.02	1.4 (0.9 to 2.4)	0.16
Days cooked QPM food specifically for young children					
QPM only	0.3				
QPM + targeting	0.5	1.8 (1.1 to 2.9)	0.02	1.6 (1.0 to 2.7)	0.06
**Panel C: Feeding and Consumption**					
Index child consumed any QPM in last 7 days‡					
QPM only	50.7%				
QPM + targeting	58.3%	1.4 (1.0 to 2.2)	0.07	1.2 (0.8 to 1.8)	0.47
Days index child consumed QPM last week					
QPM only	2.9				
QPM + targeting	3.2	1.1 (0.9 to 1.3)	0.28	1.0 (0.9 to 1.2)	0.72
Index child consumed porridge with QPM last week‡					
QPM only	26.6%				
QPM + targeting	35.0%	1.6 (1.1 to 2.3)	0.02	1.3 (0.9 to 2.0)	0.14
Days index child consumed porridge with QPM last week					
QPM only	0.5				
QPM + targeting	0.6	1.4 (1.0 to 1.9)	0.02	1.3 (1.0 to 1.7)	0.09
Index child ate from own plate‡					
QPM only	52.3%				
QPM + targeting	56.4%	1.3 (0.9 to 1.9)	0.18	1.2 (0.8 to 1.9)	0.28

Notes: OR = odds ratio for indicator outcomes from logistic regression; IRR = incidence rate ratio for days outcomes from Poisson regression; CI = confidence interval; QPM = Quality Protein Maize. *Questions are only asked in cases where the household had QPM remaining at follow-up 2. Coefficients from ordinary least squares models are reported. Partially adjusted models only control for kebele to account for stratification and are clustered at the community health group level; adjusted models additionally control for household, caregiver and index child characteristics shown in Table 1. ‡Only items marked with '‡' are in the overall compliance measure; the effect on overall compliance is estimated using OLS only and therefore excluded from this table.

Table A7. Poisson regressions of impact of the intervention package on food group consumption

		Partially adjusted		Adjusted	
	**Mean**	**IRR (95% CI)**	**p-value**	**IRR (95% CI)**	**p-value**
**Panel A: Follow-up 1**					
Number of days index child consumed conventional maize in last week					
QPM only	4.6				
QPM + targeting	4.2	0.9 (0.8 to 1.0)	0.07	0.9 (0.8 to 1.0)	0.17
Number of days index child consumed QPM in last week					
QPM only	3.6				
QPM + targeting	4.5	1.3 (1.1 to 1.4)	<0.01	1.2 (1.1 to 1.4)	<0.01
Number of days index child consumed meat or fish in last week					
QPM only	0.5				
QPM + targeting	0.5	0.9 (0.7 to 1.3)	0.75	1.0 (0.7 to 1.4)	0.82
Number of days index child consumed eggs in last week					
QPM only	0.7				
QPM + targeting	0.6	1.0 (0.7 to 1.3)	0.74	0.9 (0.7 to 1.2)	0.46
Number of days index child consumed legumes in last week					
QPM only	4.5				
QPM + targeting	4.5	1.0 (0.9 to 1.1)	0.65	1.0 (0.9 to 1.1)	0.79
Number of days index child consumed milk in last week					
QPM only	1.8				
QPM + targeting	1.8	1.0 (0.8 to 1.3)	0.74	1.0 (0.8 to 1.2)	0.80
**Panel B: Follow-up 2**					
Number of days index child consumed conventional maize in last week					
QPM only	5.1				
QPM + targeting	4.9	0.9 (0.9 to 1.0)	0.23	1.0 (0.9 to 1.1)	0.73
Number of days index child consumed QPM in last week					
QPM only	2.9				
QPM + targeting	3.2	1.1 (0.9 to 1.3)	0.28	1.0 (0.9 to 1.2)	0.72
Number of days index child consumed meat or fish in last week					
QPM only	0.5				
QPM + targeting	0.5	1.2 (0.9 to 1.7)	0.24	1.2 (0.8 to 1.6)	0.40
Number of days index child consumed eggs in last week					
QPM only	0.7				
QPM + targeting	0.6	1.0 (0.8 to 1.3)	0.96	0.9 (0.7 to 1.3)	0.68
Number of days index child consumed legumes in last week					
QPM only	4.8				
QPM + targeting	4.9	1.0 (0.9 to 1.1)	0.83	1.0 (0.9 to 1.1)	0.52
Number of days index child consumed milk in last week					
QPM only	2.2				
QPM + targeting	2.2	1.0 (0.8 to 1.3)	0.84	1.0 (0.8 to 1.2)	0.76

Notes: IRR = incidence rate ratio from Poisson regression; CI = confidence interval. Coefficients from ordinary least squares models are reported. Partially adjusted models only control for kebele to account for stratification and are clustered at the community health group level; adjusted models additionally control for household, caregiver and index child characteristics shown in Table 1.

Table A8. Ex-post minimum detectable effect (MDE) for primary outcomes

Primary outcome (measured at follow-up 1)	Partially adjusted effect estimate	Standard error (SE)	MDE (2.8*SE)
QPM grain unmixed during storage (%)	42.8	3.8	10.6
QPM flour unmixed during storage (%)	48.0	4.0	11.1
Not sold QPM since beginning of season (%)	4.3	1.9	5.4
Cooked QPM food specifically for young children (%)	15.0	3.1	8.5
Days cooked QPM food specifically for young children	0.6	0.1	0.3
Index child consumed any QPM in last 7 days (%)	19.3	3.6	10.1
Days index child consumed QPM last week	1.0	0.2	0.7
Index child consumed porridge with QPM last week (%)	24.8	3.9	11.0
Days index child consumed porridge with QPM last week	0.5	0.1	0.3
Index child ate QPM for more days than household head (%)	13.0	2.6	7.2
Difference in number of days QPM consumed between index child and household head	0.6	0.1	0.3
Index child ate from own plate (%)	7.1	4.1	11.3

Notes: MDEs are reported by calculating 2.8 times the standard error estimated from the partially adjusted regression (i.e., adjusted for kebele2 fixed effects and clustering) in order to understand MDEs at 80% power. 2.8 is the sum of 1.96 (for 5% significance level) and 0.84 (for 80% power).
